# Defending the Body Without Sensing the Body Position: Physiological Evidence in a Brain-Damaged Patient With a Proprioceptive Deficit

**DOI:** 10.3389/fpsyg.2018.02458

**Published:** 2018-12-04

**Authors:** Carlotta Fossataro, Valentina Bruno, Patrizia Gindri, Francesca Garbarini

**Affiliations:** ^1^MANIBUS Laboratory, Psychology Department, University of Turin, Turin, Italy; ^2^San Camillo Hospital of Turin, Turin, Italy

**Keywords:** defensive peripersonal space, proprioception, hand blink reflex, brain-damaged patient, body position

## Abstract

The ability to know where our body parts are located in space (proprioception) is fundamental for both successfully interacting with the external world and monitoring potential threats. In this case-control study, we investigated whether the absence of proprioceptive signals may affect physiological defensive responses. To this aim, a right brain-damaged patient with a left upper-limb proprioceptive deficit (P+ patient) and age-matched healthy controls, underwent the recording of the Hand-Blink Reflex (HBR). This defensive response, elicited by electrical stimulation of the median nerve and recorded from the orbicularis oculi, is modulated by the hand position: it is enhanced when the threatened hand is near to the face, inside the defensive peripersonal-space (DPPS). According to the classical neuropsychological perspective, we used P+ patient as a model to investigate the role of proprioception in HBR modulation, by manipulating the congruity/incongruity between the intended and actual positions of the stimulated hand. P+ patient, with his eyes closed, had to voluntarily place his left hand either far from or near to his face and to relieve the arm’s weight over a supporting device. Then, in congruent conditions, the hand was stimulated in the actual (intended) position. In incongruent conditions, the patient’s hand was moved by the examiner from the intended to the opposite (not-intended) position and then stimulated. We observed an inverse response pattern between congruent and incongruent conditions. In congruent conditions, P+ patient showed an HBR enhancement in near compared to far position, comparable to that found in healthy controls. This suggests that, even in absence of proprioceptive and visual information, the HBR modulation was still present. Conversely, in incongruent conditions, P+ patient showed a greater HBR magnitude for far position (when the hand was actually far, but the patient intended it to be near) than for near position (when the hand was actually near, but the patient intended it to be far). This result suggests that proprioceptive signals are not necessary for HBR modulation to occur. It relies more on the intended than on the actual position of the hand. The role of motor intention and planning in shaping the DPPS is discussed.

## Introduction

The development of a coherent representation of one’s own body in space requires the combination of motor commands (Burin et al., 2015, 2017; della Gatta et al., 2016; Fossataro et al., 2018), somatosensory information coming from the body (Longo et al., 2010; Serino and Haggard, 2010; Longo and Haggard, 2012; Zeller et al., 2014; Zeller et al., 2016), including both proprioceptive (Paillard, 1999; Longo et al., 2009; Longo and Haggard, 2010) and vestibular (Lopez, 2016) input, and visual information coming from both the body parts itself and the space directly surrounding the body, i.e., the so-called peripersonal space (PPS) (Makin et al., 2008; Macaluso and Maravita, 2010; Noel et al., 2018). The PPS (for a recent review see Hunley and Lourenco, 2018), from a functional point of view, can be defined either as the space within which we can interact with the environment, performing goal-directed actions (Rizzolatti et al., 1997), or as a safety margin within which we have to protect our bodies (de Vignemont and Iannetti, 2015). In this study, we focus on the latter concept, namely that of the defensive peripersonal space (D)PPS (Cooke and Graziano, 2003; Graziano and Cooke, 2006).

Since external stimuli signaling potential threats may suddenly approach the body, a continuous assessment of environmental threats according to a body reference frame, is essential to survive. The closer the threats occur, the more likely they are to cause damage. Consequently, defensive and avoidance reactions are stronger. Thus, the boundary of the DPPS can be described by looking at the amplitude of defensive responses to aversive stimuli, occurring at different locations with respect to the body. In monkeys, the electrical stimulation of the ventral intraparietal area (VIP) and a polysensory zone (PZ) in the precentral gyrus, evokes defensive responses. It is as if the monkeys are defending the body part where the sensory receptive field of the neuron is located (Graziano et al., 2002). This, added to the fact that the firing rate of the multimodal neurons in both areas (Rizzolatti et al., 1981a,b; Graziano and Gross, 1993; Fogassi et al., 1996; Duhamel et al., 1998; Graziano, 1999; Graziano et al., 2000) increases as more visuo-tactile stimuli approach the face, leads to propose that such a fronto-parietal network participates in the construction of a margin of safety around the body and in the selection of defensive behavior (Cooke and Graziano, 2003; Graziano and Cooke, 2006). In humans, the DPPS surrounding the body has been described by recording a defensive blink reflex (hand blink reflex - HBR) elicited by electrical stimulation of the median nerve. The HBR is an entirely subcortical defensive reflex and it is known to be modulated by the hand position in space. It is significantly enhanced when the threatened hand is located close to the face, inside the DPPS (Fossataro et al., 2016a,b; Sambo et al., 2012a,b).

It has been demonstrated that, in order to modulate the HBR, online visual information is not necessary. Indeed, when the hand is stimulated near to the face, the response enhancement occurs even in healthy subjects with their eyes closed (see experiment 2 in Sambo et al., 2012b) and in individuals with late-onset blindness. The absence of the HBR modulation in an individual with early-onset blindness, if confirmed in a larger sample, might suggest that the ability to modulate the magnitude of the HBR depends on the presence of a functioning visual system during early childhood (Wallwork et al., 2017). Thus, in healthy and in late-onset blindness subjects, what appears to be prioritized when modulating the HBR is the proprioception (also known as position sense); i.e., the ability to sense the body segment position and movements in space, based on sensory signals provided to the brain from muscles, joints and skin receptors (Gandevia et al., 2002; Stillman, 2002; Proske and Gandevia, 2012; Han et al., 2016; Tuthill and Azim, 2018). However, recent evidence (Bisio et al., 2017) investigating the HBR during voluntary movements shows that, irrespective of the hand position, the response enhancement was present only when the hand approached (and not receded from) the DPPS of the face (for a different study investigating the HBR during voluntary movement see Wallwork et al., 2016). Thus, when the hand is close to the face but the subject is planning to move the hand far away, the response is not enhanced. This means that, during movements, the HBR enhancement relies not only on proprioceptive (afferent) signals of the hand position, but also on motor intention and planning (efferent signals), which are able to predict the final position of the hand.

In healthy individuals, intentional outflow and somatosensory inflow (i.e., proprioceptive inputs) are both available to estimate the final position of the hand (Bisio et al., 2017). Thus, in the present study, we adopted a neuropsychological perspective and we used a pathological single case, in which movement, planning and execution were preserved while proprioception was selectively damaged (P+ patient). In this way, we were able to investigate the relative roles of these complementary sources of information in modulating the HBR amplitude.

**FIGURE 1:**
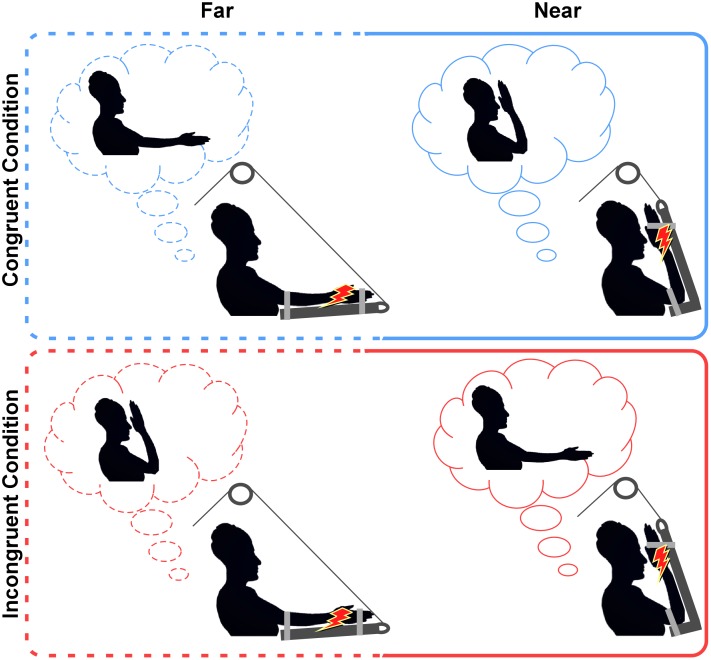
Experimental conditions. In both congruent and incongruent conditions, the P+ patient was asked to voluntary reach either far or near positions (requested positions in the light-blue and red clouds). Once the patient reached the requested position, he had to relieve the arm’s weight on the supporting device and wait for the stimulus. Note that in congruent conditions (**top panel**), the intended position and the actual position of the hand were the same, while, in the incongruent conditions (**bottom panel**) they were the opposite. Indeed, before the stimulus was delivered, the co-experimenter activated the pulley to move the patient’s arm in the opposite position. The red lightning represents the electrical stimulation delivered to the median nerve.

In our experimental design, healthy participants underwent the classical paradigm in which the HBR was recorded in both far and near positions [i.e., when the stimulated hand is placed far from and near to the face, respectively (Fossataro et al., 2016a,b; Sambo et al., 2012a,b)]. The P+ patient underwent an *ad hoc* paradigm devised to investigate the role of proprioception in the HBR modulation, by manipulating the congruity/incongruity between the intended and the actual position of the stimulated hand (see details in Materials and Methods and in Figure 1). The crucial aspect of this experiment is that, in incongruent conditions, the P+ patient, due to the proprioceptive deficit (and to the absence of visual feedback), was not able to detect the mismatch between the intended (e.g., near to the face) and the actual (e.g., far from the face) positions of the hand. If proprioception is necessary for the HBR modulation to occur, no HBR modulation should be expected in the P+ patient, irrespective of the congruity/incongruity between the intended and the actual positions of the hand. On the contrary, if the HBR modulation relies more on the intended than on the actual position of the hand, the HBR enhancement should be observed also when the stimulated hand is actually far, but the P+ patient (deprived of the visual and proprioceptive information) believes it to be near to the face (i.e., incongruent condition).

## Materials and Methods

### Case Description

The P+ patient was a right-handed (Oldfield, 1971), seventy-two years old man. In January 2017, he suffered from a right ischemic stroke and was admitted to San Camillo Hospital in Turin, for a neurorehabilitation program. He had no previous history of psychiatric disorders. Before starting the experimental procedures, he provided written informed consent to participate in the study, which was designed in accordance with the Declaration of Helsinki (BMJ 1991; 302: 1194) and approved by the Ethical Committee of the ASL TO 1 of Turin. Written informed consent was obtained from the P+ patient for the publication of this case report and their indirectly identifiable information. At the time of this study (March 2017), he was assessed using common neuropsychological tests (see Table 1 for details). At the evaluation, he appeared awake, cooperative and with an adequate mood level. Visual, motor and tactile functions were evaluated by the responsible neurologist and by a standardized neurological protocol. In this protocol, scores range from 0 (no deficit) to 3 (severe deficit) and are carried out in accordance with the procedure outlined in previous studies (e.g., Bisiach et al., 1986; Spinazzola et al., 2008; Pia et al., 2014a,b; Piedimonte et al., 2015, 2016). According to these evaluations, the P+ patient did not show signs of hemianopia (Bisiach et al., 1986) or a contralesional upper limb motor deficit (i.e., he was able to perform both distal and proximal movements requested by the examiner with the contralesional upper limb). However, he showed signs of extra-personal neglect [assessed by means of the Diller Letter H Cancellation Test (Diller and Weinberg, 1977) and the Behavioral Inattention Test – BIT (Wilson et al., 1987)], personal neglect [assessed by means of the Fluff Test (Cocchini et al., 2001)] and of tactile extinction (di Pellegrino et al., 1997; Làdavas et al., 2000). What is more crucial for this study, is that he showed a selective proprioceptive impairment of the contralesional upper limb. Proprioception, as in previous studies (Fossataro et al., 2016a, 2017; Fossataro et al., 2017), was assessed by means of two techniques for testing the limb localization: the Contralateral Limb Matching Task (CLMT) (Lincoln et al., 1991; Piriyaprasarth et al., 2009; Goble, 2010) and the Finger Localizing Test (FLT) (Head and Holmes, 1911; Lincoln et al., 1991; Hirayama et al., 1999). Both procedures were performed with the patient’s eyes closed. It should be noted that prior to the test, we instructed the patient in how to complete the task to ensure the correct performance of it (i.e., with his eyes open). In the CLMT, the patient’s affected contralesional arm was passively moved in a reference joint angle (i.e., target position) by the examiner and the patient was asked to recreate such a target position by matching it with the contralateral intact arm. In the FLT, the examiner positioned the patient’s contralesional affected limb (fixed limb) and asked him to pinch a target finger of that limb with the thumb and index finger of the opposite hand (reaching limb). For the CLMT, a correct response was designated when the difference of joint angles between the affected limb and the unaffected limb was less than 10° as judged by visual estimation. While a difference of more than 10° was designated as an incorrect response. For the FLT, a correct response was designated when the patient reached the target finger with reasonable accuracy. While an incorrect response was assigned when the patient was unable to reach the target finger. Each test consisted of a total of 10 trials. The score was calculated as percentage of accuracy. It should be noted that when the P+ patient had to reproduce (CLMT test) or to reach (FLT test) the position of the affected limb with the intact limb, his performance was never correct (0% of accuracy). Therefore, his proprioception was considered impaired. However, because of the patient’s spared motor ability, one of the two tests (namely the FLT) was also performed on the ipsilesional side of the body. That is, the patient’s ipsilesional intact limb (fixed limb) was passively moved by the examiner and the patient was asked to pinch it with the affected hand (reaching limb). In this case, the patient’s performance was always correct (100% of accuracy) (see Discussion).

**Table 1:** Patient’s demographic and clinical data.

Patient’s neuropsychological assessment
Sex	M
Education	5
Etiology	I
Lesion side	RH
Month from onset	2
General cognitive impairment	-
Visual field defect	-
Hemiplegia (HP)	-
Hemianesthesia (HA)	-
Tactile extinction	+
Proprioception	+
Extra-personal neglect	+
Personal neglect	+

*Sex: M, male. Education: years of school. Etiology: I, ischemia. Lesion side: RH, right hemisphere. Months from onset: number of months between the onset of the disease and the assessment. Presence/absence of deficit (+/-). General cognitive impairment: MOCA = 19 [cut-off ≥ 17 (Bosco et al., 2017)]. For visual field defect (for the upper and lower visual quadrants, respectively), hemiplegia, and hemianesthesia scores were ranged from normal (0: -) to severe defects (3: +). Please note that a score of one is assigned to participants who omitted the left contralesional stimulus during bilateral stimulation, but showed spared tactile sensibility when unilateral tactile stimuli were delivered to contralesional hand (Bisiach et al., 1986; Pia et al., 2014a,b). Proprioception was assessed by means of the CLMT (Lincoln et al., 1991; Piriyaprasarth et al., 2009; Goble, 2010), whereby the patient is asked to recreate (i.e., match) a reference joint angle (i.e., target position) in the absence of vision (i.e., using proprioceptive information) and by the FLT (Head and Holmes, 1911; Lincoln et al., 1991; Hirayama et al., 1999), whereby the patient is asked to reach a target finger of the contralesional arm. Extra-personal neglect: BIT (Wilson et al., 1987), conventional subtests = 53 (cut-off ≥ 129/146); BIT behavioral subtest = 28 (cut-off ≥ 67/81); DILLER Letter H Cancellation (Diller and Weinberg, 1977) =28 (cut-off omissions L–R ≤ 5). Fluff Test = 6 (cut-off omissions ≤ 2) (Cocchini et al., 2001)*.

### Lesion Mapping

The P+ patient’s brain lesion was identified by CT, and through a computerized technique, was mapped onto the MNI stereotactic space using standard MRI volume (voxels of 1 mm^3^). Free MRIcron software (Rorden and Brett, 2000) was used to perform lesion reconstruction. Firstly, the MNI template was rotated on coronal, sagittal and horizontal planes according, to the patient’s scan angle. Secondly, a skilled rater (VB) manually mapped the lesion onto each correspondent template slice, while a second skilled rater (CF) double-checked for the accuracy of the tracings. Thirdly, the maps were back-rotated into the standard space. Gray matter involvement was obtained by superimposing the Anatomical Labeling Map Template AAL (Tzourio-Mazoyer et al., 2002) and the JHU-White Matter Template (Hua et al., 2008), which categorize the distributions of digital images onto stereotactic space.

### Lesion Description

**FIGURE 2:**
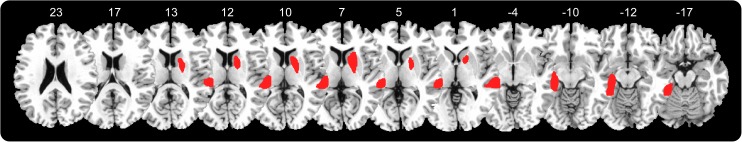
Patient lesion mapping, right hemisphere lesion involving: superior and middle temporal gyrus, basal ganglia (caudate nucleus, putamen, and pallidum), anterior limb of the internal capsule, anterior corona radiate, and external capsule.

The P+ patient presented a widespread right temporal cortical-subcortical lesion involving the right middle cerebral artery territory. The lesion mainly involved the superior and middle temporal gyrus, the basal ganglia (caudate nucleus, putamen, and pallidum), the anterior limb of the internal capsule, the anterior corona radiata and the external capsule (see Figure 2).

### Control Group

Twelve age-matched healthy subjects (7 females, mean age ± SD: 68.9 ± 4.06), who were all right-handed (Oldfield, 1971), were engaged in the experiment as a control group. All participants were naive to the experimental procedure and to the purpose of the study and provided written informed consent to participate in the study. In accordance with the Declaration of Helsinki (BMJ 1991; 302: 1194), all the experimental procedures were approved by the Ethical Committee of the University of Turin and by the Ethical Committee of the ASL TO 1 of Turin.

### Experimental Paradigm

Before starting the experiment, the P+ patient was instructed about the postural manipulation that was to be performed during the experiment. He had to place the left contralesional hand either far from the face (i.e., at a distance of ∼60 cm from the face) or near to the face (i.e., at a distance of ∼4 cm from the ipsilateral side of the face). In each trial, the experimenter asked the patient to reach one of the two positions (far; near) and the patient voluntarily performed the postural manipulation. Then, he had to keep the arm relaxed, unloading the arm’s weight over a dedicated device able to sustain it, and to wait for the electrical stimulus delivered to the median nerve (see details in Stimulation and Recordings). The device was specifically built to fix the patient’s forearm over a soft panel and sustain the elbow by means of an adjustable extension able to completely support the arm’s weight. The device was connected to a pulley, which allowed the co-experimenter to move the patient’s arm in space. The postural manipulation was performed in two different conditions, depending on the congruity/incongruity between the intended and the actual positions of the hand. According to the condition, the supporting device was kept in the same position requested to the patient (congruent condition) or moved by the co-experimenter in the opposite one (incongruent condition); see Figure 1. This leads to a 2^∗^2 factorial design, with four experimental conditions, namely “far-congruent” and “near-congruent,” in which the intended position and the actual positions of the hand were the same; “far-incongruent” and “near-incongruent,” in which the intended position and the actual position of the hand were the opposite. The study consisted of four blocks where experimental conditions were presented in a counterbalanced order (i.e., congruent, incongruent, incongruent, congruent, congruent, incongruent, incongruent, and congruent). Each block lasted for 3 min and consisted of a total of 6 trials; 3 in the far position and 3 in the near position in alternating trials, resulting in a total of 24 trials (12 far and 12 near) per condition. It is important to note that, for the purpose of the study (i.e., investigating the role of proprioceptive signals in modulating the HBR), visual information was precluded. Thus according to a previous study (see experiment 2 in Sambo et al., 2012b), demonstrating that HBR enhancement within the DPPS of the face can be recorded irrespective of the presence or absence of online visual feedback, both the patient and controls were asked to keep their eyes closed for the duration of each experimental block (3 min), including both postural manipulation and HBR recording.

Healthy participants underwent the classic HBR paradigm, in which the stimulated hand was voluntarily placed either far from or near to the face (Sambo et al., 2012a,b; Fossataro et al., 2016a,b). In the same way, as in the patient’s congruent condition, they had to relieve the arm’s weight over the supporting device and to wait for the stimulus.

### Stimulation and Recordings

Electromyographic activity (EMG) was recorded from the orbicularis oculi muscle bilaterally, using two pairs of bipolar surface electrodes. The active electrode was placed over the mid lower eyelid and the reference electrode laterally to the outer canthus. EMG signals were amplified and digitized at 10 kHz (BIOPAC System, MP150), and stored for offline analysis. The HBR was elicited by delivering transcutaneous electrical stimuli to the left median nerve, using a surface bipolar electrode (constant current square-wave pulses; DS7A, Digitimer). In each participant, by increasing the stimulus intensity until a clear and stable HBR was observed in at least five consecutive trials or the participant refused a further increase in intensity, we calibrated the stimulus intensity able to elicit a clear blink reflex (mean stimulus intensities, 33.75 ± 10.32 mA; range, 20–56 mA). The stimulus duration was 200 μs and to minimize stimulus habituation the inter-stimulus interval was ∼30 s, following prior methods (Sambo et al., 2012a,b; Sambo and Iannetti, 2013; Fossataro et al., 2016a,b; Bisio et al., 2017).

### Data Pre-processing

Electromyographic activity pre-processing and analysis were completed using MATLAB^1^ and Letswave^2^ software (Mouraux and Iannetti, 2008). EMG signals from each participant were high-pass filtered (55 Hz), full-wave rectified and then averaged across ipsilateral and contralateral recording sides. HBR responses were averaged separately according to condition: in the P+ patient, resulting in four waveforms (far-congruent; near-congruent; far-incongruent; near-incongruent); in healthy subjects, resulting in two waveforms for each subject (far-congruent and near-congruent). We extracted and measured the area under the curve (AUC) of the average HBR waveform for each subject and condition.

### Data Analysis

In the healthy subjects group, we compared the HBR magnitude between far-congruent and near-congruent condition by means of a paired *t*-test (2 tailed). Then, an index of the HBR modulation, calculated as the difference between hand positions (Δ near-far), was used as a dependent variable to perform case-control comparisons by means of the Crawford’s test (Crawford et al., 2010; Fossataro et al., 2017; Garbarini et al., 2018). This test is specifically devised to test whether an individual’s score is significantly different from a control sample and provides a point and interval estimate of the abnormality of the case’s score (i.e., it estimates the percentage of the population that would obtain a lower score, together with a 95% confidence interval on this percentage). Note that two different comparisons were performed between the P+ patient and controls. In the first comparison, we used the P+ patient’s HBR modulation index in the congruent conditions. While in the second comparison, the P+ patient’s HBR modulation index in the incongruent conditions was used.

## Results

In the healthy subjects group, as expected, the paired *t*-test revealed a greater HBR magnitude in the near compared to the far positions [*t*_(11)_ = 6.28; *p* = 0.0001; power = 0.99; HBR magnitude: mean ± SD; far: 11.97 ± 8.93; near: 14.57 ± 10.06].

In the case-control comparisons, the Crawford’s test revealed no significant differences between the P+ patient’s congruent conditions and controls; i.e., the P+ patient showed an HBR enhancement in the near compared to the far condition, comparable to that found in the healthy controls (*t* = 0.36; *p* = 0.36; Z-CC = 0.38; 95% confidence interval = -0.215 to 0.959; mean ± SD Δ near-far congruent controls = 2.59 ± 1.58; Δ near-far congruent P+ patient = 3.19).

**FIGURE 3:**
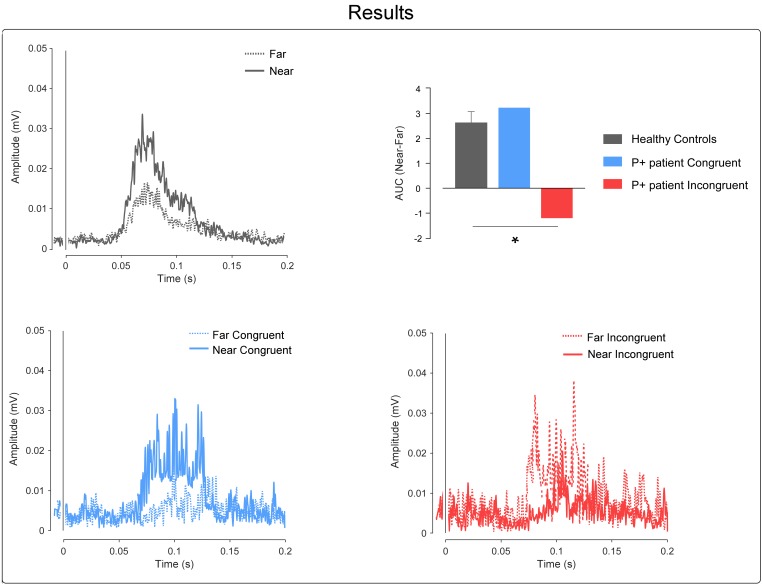
Results. **Top-left**, the graph shows the control’s group-average rectified HBR waveforms; *x*-axis, Time (ms); *y*-axis EMG activity (mV). **Top-right**, the graph shows the AUC expressed as a difference between the near and far positions; in the controls group, we plotted the mean group AUC and error bar representing the standard error of the mean (SEM). The asterisk represents the results of the Crawford’s test (^∗^ = *p* < 0.05). **Bottom**, the graphs show the P+ patient’s rectified HBR waveforms in the congruent and incongruent conditions, respectively; *x*-axis, time (ms); *y*-axis EMG activity (mV).

On the contrary, the Crawford’s test revealed significant differences between the P+ patient’s incongruent conditions and controls; i.e., the P+ patient showed an opposite response pattern in comparison to the controls, with greater HBR values for the far position (when the hand was actually far, but the patient intended it to be near) than for the near position (when the hand was actually near, but the patient intended it to be far) (*t* = -2.27; *p* = 0.02; Z-CC = -2.36; 95% confidence interval = -3.482 to -1.227; mean ± SD Δ near-far congruent controls = 2.59 ± 1.58; Δ near-far incongruent P+ patient = -1.15); see Figure 3.

## Discussion

The present study focused on the role of proprioception in modulating defensive responses to threatening stimuli, occurring close to our body, within the DPPS. To this aim, we took advantage of a physiological mechanism, namely the HBR, known to be modulated by the position of the stimulated hand in space (Sambo et al., 2012a,b; Bufacchi et al., 2015; Fossataro et al., 2016a,b). In adopting a neuropsychological perspective, we used a brain-damaged patient with spared motor abilities and a selective proprioceptive deficit of the contralesional upper limb (P+ patient), as a model to investigate the role of efferent and afferent sources of information in modulating the HBR amplitude. If the HBR enhancement mainly relies on afferent signals of the hand position, then, in the P+ patient, the absence of proprioceptive signals would suppress the HBR enhancement when the hand was stimulated near to the face. Alternatively, our results suggest that the HBR enhancement relies more on motor intention and planning (efferent signals), able to predict the hand position, than on proprioceptive feedback (afferent information).

In the healthy control subjects, our results confirm the previously described “hand position” effect, which suggest that the HBR is significantly enhanced by approximately a factor of two, when one’s own stimulated hand enters the DPPS of the face (Sambo et al., 2012a,b; Sambo and Iannetti, 2013; Fossataro et al., 2016a,b; Bisio et al., 2017). It has been suggested that such enhancement may result from the modulation of the excitability of the brainstem circuits, which mediates the HBR. This modulation is exerted by associative cortical areas, involved in the PPS representation and in the detection of potentially dangerous stimuli near the face (Sambo et al., 2012a,b). We also confirm that, in order for the HBR modulation to occur, proprioceptive information can be sufficient, as previously proved by the significant HBR modulation even in absence of visual information in both late-onset blindness individuals (Wallwork et al., 2017) and healthy subjects with their eyes closed (Sambo et al., 2012b). Interestingly, our data also suggest that, in healthy control subjects, the tonic contraction of the arm muscles, to maintain the hand near to the face, does not play a role in the HBR modulation [see also (Fossataro et al., 2016a; Bisio et al., 2017)]. Indeed, our design implied that participants, after having performed the postural manipulation voluntarily (placing the hand either far from or near to the face), had to relieve the arm’s weight over a supporting device and had to wait for the stimulus.

The P+ patient represents a perfect model for further investigating the role of motor intention and planning (efferent information) versus sensorimotor feedback (afferent information) in shaping the HBR magnitude. This is a rare case of a patient with spared tactile and nociceptive sensibility (i.e., he had no hemianesthesia) and spared motor ability (i.e., he had no hemiplegia), but with a selective deficit of proprioception. Indeed, even if he could perceive the electrical stimuli delivered to the median nerve and he was able to verbally report that the electrical stimulation occurred on his left wrist, he was not able to use this somatosensory information to localize the position of the affected hand. Thus, according to the classical dichotomy between body image (i.e., cognitive representation of the body based on memory stores) and body schema (i.e., a sensorimotor map based on ongoing proprioceptive inputs) (Head and Holmes, 1911; Gallagher, 1986; Paillard, 1999; Dijkerman and de Haan, 2007; Anema et al., 2009), we can say that the P+ patient had a spared body image and an affected body schema. Furthermore, due to the spared motor system, he could voluntarily perform the postural manipulation with the contra-lesional arm/hand during the HBR recording, even if, due to the proprioceptive deficit (and to the absence of visual feedback), he was not able to detect the mismatch between the intended and the actual positions of the hand during incongruent conditions. It is worth noting that, during the far/near postural manipulations, the P+ patient correctly achieved the requested position, even if he was not able to localize the affected hand in space and visual feedback was prevented for the duration of the experimental block. This could be explained by the fact that, in the near position, the arm movement was directed toward a body part, namely the patient’s head. This may have served as a frame of reference for orienting the movement. Interestingly, with the P+ patient, a spared ability to perform self-directed movements was also suggested by the clinical evaluation of the proprioceptive deficit. When, during the FLT task, the patient was requested to use his affected hand to reach for his intact hand (passively displaced by the examiner), he correctly performed the task. This is probably because information about the position of the other parts of his body may have been able to correctly orient the movement. Furthermore, it has been proposed that even the sensory signals centrally produced by the internal forward model [which estimates the sensory consequences of the movements based on the efference copy of this motor command (Wolpert and Ghahramani, 2000)], may play an additional role with respect to proprioceptive and visual signals in action execution (Farrer et al., 2003). The case of a patient able to correctly perform complex motor tasks, despite the absence of visual feedback and the impairment of proprioceptive input, supports such a view (Fourneret et al., 2002).

From an anatomical point of view, the P+ patient’s brain lesion is compatible with the findings of previous literature about the proprioceptive loss in stroke patients and the neural correlation of position sense in healthy participants. With respect to the basal ganglia, they are traditionally related to a series of motor functions, including an important role in intentional motor programming, known to exert a modulatory effect on the motor cortex activity (Grillner and Robertson, 2016). Certainly, basal ganglia dysfunction has usually been associated with several movement disorders (Nambu, 2008; Obeso et al., 2014). However, this motor function seems to not be affected in our patient, who showed a spared motor ability. More coherently with the P+ patient’s clinical evaluation, basal ganglia lesions may lead to the proprioceptive deficit. The role of basal ganglia in processing proprioceptive feedback has been described (Lidsky et al., 1985) in both pathological contexts (Maschke et al., 2003) and neuroimaging studies in healthy subjects. In two case reports (Halligan et al., 1993; Kim et al., 2017), the described patients, with basal ganglia damage, showed sensory and proprioception impairment, including disturbances in the normal experience of body schema (i.e., supernumerary phantom limb). In healthy participants, neuroimaging studies performed during muscle tendon vibration, known to alter the proprioceptive feedback by inducing a kinesthetic illusory limb movement (Goodwin et al., 1972), identified subcortical activation within the basal ganglia, including the putamen (Naito et al., 2005, 2007). Accordingly, a recent study (Goble et al., 2012), which investigates the proprioceptive decline in elderly, identified subcortical activity in basal ganglia (pallidum and putamen) with significantly reduced levels of activity in the right putamen, when comparing older individuals with younger ones. Additionally, from single-cell recording studies involving monkeys, we know that neurons in the putamen (Crutcher and DeLong, 1984) and in the globus pallidus (DeLong et al., 1985), code for passive joint rotation. However, in the P+ patient, other lesions may also account for the proprioceptive deficit, namely the anterior limb of the internal capsule and anterior corona radiate, supporting the well-known division between the anterior (ascending) somatosensory pathways (Sacco et al., 1987; Kim et al., 1992) and posterior (descending) motor pathways (Franzini et al., 2008; Jang, 2009; Kenzie et al., 2014) at the level of both the internal capsule and corona radiata.

This pathological condition, with the spared motor component and affected position sense, allowed us to describe an important new finding. As proved by the P+ patient’s results in the congruent conditions, even though proprioceptive information was lost (due to the brain lesion) and visual information was precluded (due to the patient having his eyes closed), the HBR modulation was still present. This suggests that even if proprioceptive information can be sufficient for HBR modulation to occur (see above), it is not necessary. In the P+ patient, the HBR modulation occurred even when the ability to sense the limb position was completely lost.

In the framework of proprioceptive deafferentation, other than motor intentionality, embodiment processes also assure eliciting of the HBR. An our previous study, which investigates HBR in a pathological context, suggested that HBR modulation is present in brain-damaged patients with lost position sense (Fossataro et al., 2016a). However, the focus of that study was on visual feedback, coming either from the patient’s own limb or from the examiner’s limb. In order to investigate the existence of a body-ownership dependent modulation on the HBR enhancement, we capitalized on a delusion of body ownership, recently described in brain-damaged patients. The patients systematically misidentify the other’s limb as their own, showing a pathological form of embodiment (E+ patients). Whenever the examiner’s arm/hand is located in a body-congruent position, aligned with the patient’s shoulder, E+ patients claim that this alien arm/hand is their own hand and they treat and care for it as if it was their own (Garbarini et al., 2013, 2014, 2015; Pia et al., 2013, 2016; Fossataro et al., 2016a,b; Piedimonte et al., 2016; Garbarini et al., 2017 Fossataro et al., 2017). Coherently, when the examiner performed the postural manipulation instead of the patient, the observation of the alien (embodied) hand close to the patient’s face elicited an HBR enhancement comparable to that found when a threat is brought near the face by their own hand. Note also that, in a control patient with an intact sense of body ownership (i.e., without pathological embodiment; E- patient) and with a proprioceptive deficit comparable to that shown by the E+ patients, the HBR enhancement was not present during the observation of the examiner’s arm close to the patient’s face.

In the present study, the visual feedback was precluded (i.e., both the P+ patient and healthy controls had their eyes closed), so that the HBR modulation we observed in the P+ patient could only be driven by the patient’s beliefs about the hand position, depending on the voluntary motor program implemented to reach the requested position (either up to or down from the face). This was clearly evident in the incongruent conditions, when the HBR was greater in the far position (when the hand was actually far, but the patient intended it to be near) than in the near position (when the hand was actually near, but the patient intended it to be far). Therefore, in the P+ patient, it seems that the HBR modulation relies more on the intended than on the actual position of the hand.

From an evolutionary perspective, it makes sense that a defensive response, such as the HBR, can be modulated by several different factors. Within these factors, together with primary sources of information such as vision and proprioception, motor intention and planning (informing us about the predicted position of the hand) can play a crucial role.

## Author Contributions

CF and FG designed the research and analyzed the data. CF, VB, and FG performed the research and prepared the figures. CF and VB performed patients’ lesion mapping. PG recruited patients and performed neuropsychological assessment. All authors wrote the manuscript.

## Conflict of Interest Statement

The authors declare that the research was conducted in the absence of any commercial or financial relationships that could be construed as a potential conflict of interest.

## Footnotes

1 http://www.mathworks.com

2 http://nocions.webnode.com
